# Systematic identification of conserved motif modules in the human genome

**DOI:** 10.1186/1471-2164-11-567

**Published:** 2010-10-14

**Authors:** Xiaohui Cai, Lin Hou, Naifang Su, Haiyan Hu, Minghua Deng, Xiaoman Li

**Affiliations:** 1 Center for Research in Biological Systems, University of California, San Diego, La Jolla, CA, 92093, USA; 2 School of Mathematical Sciences and Center for Theoretical Biology, Peking University, Beijing, 100871, China; 3 State Key Laboratory of Proteomics, Beijing Proteome Research Center, Beijing Institute of Radiation Medicine, Beijing, 102206, China; 4 School of Electrical Engineering and Computer Science, University of Central Florida, Orlando, FL, 32816, USA; 5 Burnett School of Biomedical Science, University of Central Florida, Orlando, FL, 32816, USA

## Abstract

**Background:**

The identification of motif modules, groups of multiple motifs frequently occurring in DNA sequences, is one of the most important tasks necessary for annotating the human genome. Current approaches to identifying motif modules are often restricted to searches within promoter regions or rely on multiple genome alignments. However, the promoter regions only account for a limited number of locations where transcription factor binding sites can occur, and multiple genome alignments often cannot align binding sites with their true counterparts because of the short and degenerative nature of these transcription factor binding sites.

**Results:**

To identify motif modules systematically, we developed a computational method for the entire non-coding regions around human genes that does not rely upon the use of multiple genome alignments. First, we selected orthologous DNA blocks approximately 1-kilobase in length based on discontiguous sequence similarity. Next, we scanned the conserved segments in these blocks using known motifs in the TRANSFAC database. Finally, a frequent pattern mining technique was applied to identify motif modules within these blocks. In total, with a false discovery rate cutoff of 0.05, we predicted 3,161,839 motif modules, 90.8% of which are supported by various forms of functional evidence. Compared with experimental data from 14 ChIP-seq experiments, on average, our methods predicted 69.6% of the ChIP-seq peaks with TFBSs of multiple TFs. Our findings also show that many motif modules have distance preference and order preference among the motifs, which further supports the functionality of these predictions.

**Conclusions:**

Our work provides a large-scale prediction of motif modules in mammals, which will facilitate the understanding of gene regulation in a systematic way.

## Background

The identification of motifs and motif modules is one of the most critical steps to understanding gene regulation. A motif, often represented by a position weight matrix, is the common pattern of short DNA segments bound by a transcription factor (TF). These DNA segments are called transcription factor binding sites (TFBSs). In high eukaryotes, including humans and mice, it is often the interplay of multiple TFBSs from different motifs, instead of from a single motif, that determines the temporal and spatial expression patterns of genes [1,2]. We define a motif module as a group of several motifs, whose TFBSs co-occur in many short DNA sequences of one kilobase (kb) long. We also define cis regulatory modules (CRMs) as 1 kb long sequences containing TFBSs of all the motifs of a motif module. Because the binding of TFs to their TFBSs plays a pivotal role in controlling gene expression and the dysfunction of these binding sites often results in diseases [3,4], it is important to identify motifs and motif modules.

Many methods are available for the identification of motifs and motif modules. Conventionally, TFBSs and motifs were identified on a gene-by-gene basis by experiments such as DNase footprinting [5] and gel-mobility shift assay [6,7]. It is through these experiments that we understand many basic principles of motifs. However, such experiments cannot address the challenging problem of identifying motifs and motif modules in the neighborhood of thousands of genes. Thus, many computational methods have been developed [2,8-20], which are often based on the following motif properties: overrepresentation, conservation, and clustering. Motif overrepresentation means that TFBSs of a motif occur in the non-coding regions of a significant number of genes. Also, TFBSs of a motif are often conserved in different species. Finally, motifs are often clustered, with multiple TFBSs of different motifs often co-occurring in short DNA regions such as CRMs. Based on these properties, previously developed computational methods have shown some success in identifying motifs and motif modules in a group of putative co-regulated genes as well as on a genome-wide scale. At the same time, new experimental technologies such as chromatin immunoprecipitation followed by microarray experiments (ChIP-chip) [21] and high-throughput sequencing of immunoprecipitated fragments (ChIP-seq) [22,23] can provide thousands of short potential TFBS residing regions for computational methods to further identify motifs and motif modules.

Although there are many methods for motif and motif module identification, methods that can handle all of the non-coding regions of the human genome are still in great need. Many computational methods were designed to identify novel motifs and motif modules only in the promoter regions of a group of co-regulated genes. These methods are successful in identifying motifs and motif modules in simple organisms such as yeast. They are not, however, as successful at identifying motifs and motif modules in higher eukaryotes such as humans. This is because the TFBSs in higher eukaryotes can be several hundred thousand base pairs (bps) upstream, downstream, or in the introns of genes. To identify TFBSs in the long non-coding regions of higher eukaryotes, several methods based on multiple genome alignments were developed [24,25]. However, current multiple genome alignments may not be able to align TFBSs and their orthologous counterparts well. For instance, multiple genome alignments from several popular methods are significantly different [26]. Although ChIP-chip or ChIP-seq experiments can narrow down potential TF targeting regions, they are still costly and limited by the availability of high quality antibodies. Thus, novel methods that can systematically identify motif modules in the entire non-coding regions around human genes are needed.

Here we describe a computational method that identifies CRMs and motif modules in the human genome. Unlike the computational methods for promoter regions, our method works on the entire non-coding sequences around human genes. The non-coding sequences of a human gene include the upstream non-coding sequence until the nearest codon of the 5' adjacent gene, the downstream non-coding sequence until the nearest codon of the 3' adjacent gene, and the intron sequences of the gene itself. Unlike all previous methods, our method measures sequence conservation based on discontiguous sequence similarity [27], which greatly expands the range of the conserved sequences. Our method is also different from the multiple genome alignment based methods, in that we use local alignments, which enables us to identify conserved TFBSs and CRMs that may be "misaligned" in the multiple alignments [26].

By applying the method to all human genes with mouse or rat orthologs in the Mouse Genome Informatics database (MGI), we have identified 3161839 motif modules, 90.8% of which are already supported by various sources of functional evidence. Compared with 14 ChIP-seq experiments, on average, our methods predicted 69.6% of ChIP-seq peaks with TFBSs of multiple TFs. Our findings also show that TFBSs of motifs in many motif modules have preferred distances and orders. All predicted motif modules are available at http://www.cs.ucf.edu/~xiaoman/module1109. We are developing a database, which will enable easier access to these predictions.

## Results

### A new method for the identification of motif modules and CRMs

The major obstacle for CRM and motif module identification in the entire non-coding regions of the human genome is the large space in which potential TFBSs may reside. For one human gene, the non-coding region can be millions of bp long. Such long non-coding regions for thousands of human genes prevent the direct application of classical statistical methods [8,15] for CRM and motif module identification, due to the convergence time of these methods in the entire human genome. The large TFBS search space also hinders the direct application of deterministic regular pattern recognition methods because almost every k-mer (a DNA segment of k bp long, k from 6 to 14 typically) is enriched. Note that CRMs, regulatory units of 1 kb long or so that contain multiple TFBSs, frequently occur in the human genome and control specific gene expression patterns [28]. Therefore, ideally, to understand gene expression patterns, we should identify these regulatory units and consider each unit independently. That is, we should select short regions that are potential CRMs from the long non-coding sequences around each gene and then only consider these selected short regions for TFBS identification. So, how to select potential CRMs without any idea of the TFBSs? Requiring the entire CRM regions to be conserved is too restrictive, perhaps only those 6-14 bp long TFBSs within the CRMs are conserved. Furthermore, the CRM regions may be shuffled in evolution and cannot be aligned with their true orthologous CRM regions in multiple genome alignments. Therefore, to identify potential CRMs, we should develop methods that do not rely on multiple genome alignments.

**Figure 1:**
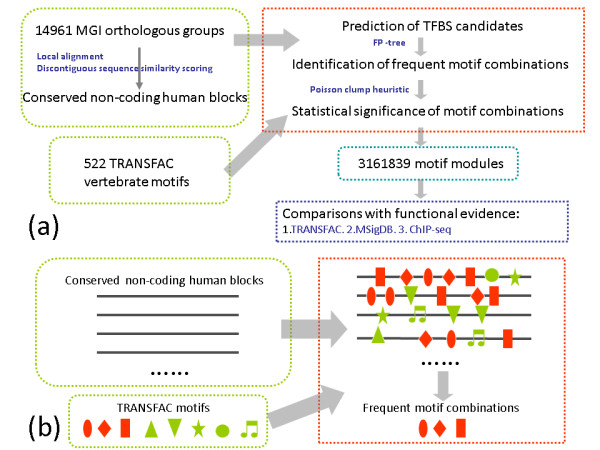
**Flow chart describing our method**. (a) The basic procedure in our method. (b) The procedure to identify motif modules from conserved blocks.

We developed such a method to systematically identify motif modules and CRMs in the entire non-coding sequences around human genes (Figure 1). In brief, we first collected all orthologous gene groups from MGI and defined the non-coding sequences of each gene based on the locations of genes in each species. Each group of orthologous genes contains one human gene and at least one mouse or rat orthologous gene. The non-coding sequences of a gene include the upstream non-coding sequence until the nearest codon of the 5' adjacent gene, the downstream non-coding sequence until the nearest codon of the 3' adjacent gene, and the intron sequences of the gene itself. Second, from the non-coding regions of every group of orthologous genes, we selected several 1 kb long "orthologous" regions based on local alignments and discontiguous sequence similarity (See Methods). We call these 1 kb long regions blocks. We used 1 kb as the block length cutoff, because 99.2% of the predicted CRMs in a genome wide study are shorter than 1 kb [24]. Third, we defined TFBS candidates by scanning the conserved segments in these blocks using 522 vertebrate motifs from the TRANSFAC database [29]. Finally, we predicted significant motif modules by applying frequent pattern tree (FP-tree) techniques [30,31] and a Poisson clump heuristic [32] to identify frequent instances of motif combinations in these blocks. In total, we predicted 3161839 motif modules and 116226 CRMs.

**Figure 2:**
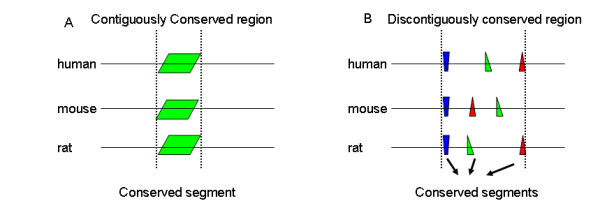
**Contiguously conserved regions and discontiguously conserved regions**. Discontiguously conserved regions often contain long divergent sequences, which makes the percent identity of the alignment of the corresponding regions be low.

Figure 2 illustrates the necessity of using discontiguous sequence similarity to define. There are two types of conserved CRMs (Figure 2). One is the CRMs that are highly conserved, such as that in Figure 2a and those defined in [33]. The other is the CRMs containing conserved segments that are interspersed by long and divergent sequences, such as that in Figure 2b. If we measure sequence conservation by considering the similarity of two regions based solely on global alignments of the two regions, the contiguous sequence similarity, we can often only identify the conserved CRMs such as the one in Figure 2a. If we measure sequence conservation by considering the similarity of pairs of conserved segments only, the discontiguous sequence similarity, we may identify both types of conserved CRMs shown in Figure 2. Note that discontiguously conserved regions do exist, and the conserved segments in discontiguously conserved regions often contain conserved TFBSs. For instance, Shashikant et al have shown a pair of functional CRMs in Hoxc8, which function in both mouse and fugu, and are composed of several short conserved segments separated by divergent sequences [34]. If we consider the contiguous sequence similarity, we would misclassify this pair of conserved CRMs, due to the fact that only 50.5% of the corresponding bps in the two CRMs are the same. Thus, to identify conserved CRMs in the human genome, we need to consider discontiguous sequence similarity, which measures conservation based on the similarity of conserved segments only.

### Comparisons with various forms of functional evidence

We compared our predictions with composite element (CE) pairs in the TRANSFAC database [29] and pre-defined gene sets in MSigDB database [35]. The comparisons show that 90.8% of the predicted motif modules are supported by functional evidence. We also compared the predicted TFBSs with the readout of 14 ChIP-seq experiments (additional files 1 and 2). With a false discovery rate (FDR) <0.01, the mean of the percentages of the predicted TFBSs within the defined ChIP-seq peaks is 10.7%, which is comparable with the previous study [24]. We compared the predicted CRMs with several large-scale CRM or enhancer studies as well, which further support our CRM and motif module predictions. In the following, we described each of these comparisons in details to justify our predictions on different levels.

We first compared the predicted motif modules with the CE pairs in TRANSFAC [29]. A CE pair contains two different motifs that are experimentally verified to provide combinatorial transcriptional regulation [36]. For the 522 vertebrate motifs we used, which can form 135981 possible motif pairs, there are 2515 CE pairs in TRANSFAC. For the predicted 3161839 motif modules, there are 21635 motif pairs, among which 528 are CE pairs. According to the hypergeometric distribution, the p-value of observing at least 528 CE motif pairs out of 21635 predicted motif pairs is 7.17E-12. Several factors, such as the number of orthologous gene groups available, the number of known motifs, and species specific CE pairs, may prevent all 2515 CE pairs from being included in the predictions.

We next compared the predicted target genes with the collected gene sets in MSigDB [35]. The predicted target genes are the closest genes to the predicted CRMs, which can be in the 5' non-coding regions, the 3' non-coding regions, or the introns of their target genes. The collected gene sets in MSigDB are based on gene annotations, known pathways, gene expression data, miRNA target genes and other factors [35]. In Table 1, for each category, we listed the number of collected gene sets and the number of motif modules with target genes significantly overlapping with these collected gene sets. It is surprising to see that the target genes of 2645699 (83.7%) of the predicted motif modules are also the target genes of miRNAs in the PicTar gene sets. MiRNAs often bind to the 3' of mRNAs of their target genes for post-transcriptional control. The observation that target genes of many motif modules are also target genes of miRNAs raises the possibility of coordinated gene regulation by TFs and miRNAs at the transcriptional and post-transcriptional level. With an FDR cutoff of 0.05 and 0.01, the target genes of 2871863 (90.8%) and 1855459 (58.7%) of the predicted motif modules significantly overlap with at least one gene set in the MSigDB database, respectively. These supported motif modules are most likely biologically meaningful. In fact, we found literature support for many motif modules. Because of page limit, we only show two examples here.

**Table 1:** Functional evidence used.

Source	Number of gene sets	Number of significant motif modules	
		
		FDR = 0.05	FDR = 0.01
BioCarta	155	0	0

KEGG	168	122,279	10,378

Genmapp	88	5486	14

GO	1141	1,296,621	460,973

PicTar	162	2,645,699	1,647,433

Cancer Module	380	485,850	67,135

Total	2094	2,871,863	1,855,459

The first column lists the different sources of functional evidence. The second column is the number of all gene sets. The third and fourth columns are the number of motif modules whose target genes significantly overlap with the gene sets. All of them except KEGG pathways are downloaded from MSigDB. For KEGG pathways, we directly downloaded from the KEGG ftp site. Only the gene sets with at least ten genes are used.

#### Example 1

The motif module "2525420" comprises motifs M00135 (OCT1), M00962 (AR), and M00342 (OCT1). AR is known to interact with OCT1 physically [37]. OCT1 is important in lens and olfactory placode development in the mouse [38] and AR is suggested to influence central nervous system development [39]. The function of the TFs in this motif module is consistent with the functions of the target genes. The 311 target genes significantly overlap with the pathway genes in KEGG: hsa04360 (axon guidance) and significantly overlap with the genes annotated with "nervous system development". Axon guidance represents a key stage in the formation of a neuronal network, which supports the functionality of TFs in this motif module.

#### Example 2

The motif module "285236" is composed of motifs M00034 (P53), M00938 (E2F-1), M00189 (AP-2), and M00982 (KROX). P53 is a well known tumor suppressor protein and involved in various types of cancer. E2F-1 is a member of the E2F family of TFs that plays a crucial role in the control of cell cycle and tumor suppressor protein activities. AP-2 is a TF that regulates proliferation and differentiation in mammalian cells, and is involved in prostate cancer development [40]. KROX, also known as EGR-1, activates genes that are required for differentiation and mitogenesis, and is over-expressed in prostate cancer [41]. Thus, these TFs may work together to play a role in prostate cancer. Consistently, the 99 target genes of this motif module significantly overlap with the target genes of miR-330. MiR-330 induces apoptosis in prostate cancer cells through E2F1-mediated suppression of Akt phosphorylation [42], which strongly supports the coordinate gene regulation by this motif module and miR-330.

Besides the comparisons with the CE pairs in TRANSFAC and the gene sets in MSigDB, we also compared the predicted TFBSs with 14 ChIP-seq experiments (additional files 1, 2, and 3). There are 30 motifs corresponding to 13 TFs used in these ChIP-seq experiments. With an FDR of 0.01, the mean and the maximal precision of our predictions, defined as the percentage of predicted TFBSs within the ChIP-seq peaks, are 10.7% and 31.9%, respectively. A previous study based on multiple genome alignments had a comparable precision of 3% and 17% in two ChIP-chip experiments, where the authors selected the predicted CRM regions as probes to make the microarray [24]. As in the previous study [24], the precision here is most likely underestimated. On the other hand, the mean and the maximal recall of our predictions, defined as the percentage of ChIP-seq peaks with predicted TFBSs among all ChIP-seq peaks with putative binding sites, are 5.0% and 17.1%, respectively. Notice that the low recall is mainly due to the fact that ChIP-seq experiments target TFBS residing regions of individual motifs while our predictions aim to identify motif combinations. In fact, if we compare the predicted TFBS pairs of two TFs with the ChIP-seq peaks containing putative TFBSs of the two TFs, the mean and the maximal recall is 69.6% and 100.0%, respectively, with an FDR of 0.01 (additional files 1 and 3). For instance, if we consider the individual motifs M00069 (YY1) and M00322 (c-myc), 10.2% and 7.1% of the ChIP-seq peaks are predicted to overlap with CRMs containing the YY1 binding sites and c-myc binding sites, respectively (additional file 2). If we consider the motif pair (M00069, M00322), 83.8% of the ChIP-seq peaks with YY1 binding sites and c-myc binding sites overlap with the CRMs containing the two types of binding sites (additional file 3).

We also compared the predicted 116226 CRMs with the 123510 pCRMs [24]. These pCRMs are computationally predicted CRMs that are significantly enriched in TFBSs for one to five different TFs within 2 kb long regions. To our knowledge, these pCRMs are the only available mammalian CRMs predicted in the entire non-coding sequences around genes based solely on sequence information and known motifs. Identified by using all known vertebrate motifs in the TRANSFAC database to scan the genome alignment of human, mouse and rat genomes, these pCRMs cover 59559857 human bps (2% of the human genome). We found that 63425 of our predicted 116226 CRMs (54.6%) overlap with the pCRMs. The remaining 45.4% of CRMs do not overlap with pCRMs, due to the genome rearrangement and/or the fact that genome alignments cannot align TFBSs well. We also found that 64972 out of 123510 pCRMs (52.6%) overlap with our predicted CRMs. For the remaining 47.4% of the pCRMs, 40.6% contain TFBSs of one or two TFs. These 19.2% (47.4%*40.6%) of the pCRMs may be too weak to be considered as putative functional regions under the schema of the discontiguous sequence similarity.

In addition to the comparison with the pCRMs, we also compared our predicted CRMs with specific enhancers from two recent studies [43,44]. Visel et al. detected 2543, 561, and 2105 p300 (an enhancer-associated protein) peaks in mouse embryonic forebrain, midbrain and limb tissue, respectively [44]. They mapped 86 of these sequences to homologous human regions and found that 75 of the human regions had enhancer activity. We found 61 out of these 75 (81.3%) enhancers are included in our predicted CRMs. Narlikar et al. selected 41930 human sequences as putative heart enhancers by using known motifs and gene expression data [43]. We found 13895 out of the 41930 (33.1%) predicted enhancers overlap with our predicted CRMs. Several factors affect the percentage of overlapping with the predicted enhancers in the second study, such as the number of orthologous genes available in our study, the expression data and prior biological knowledge used in the second study, the false positives in each study. Note that published studies on CRMs often require the occurrence of multiple TFBSs in short regions in order to claim these regions to be CRMs, while we require both the occurrence of multiple TFBSs in short regions and the occurrence of the same motif combinations in many short regions. Our requirement of recurrent occurrence of motif combinations may enable us to filter a significant portion of false positives from our predictions.

### The order and distance preference of motif modules

The order of the TFBSs of different TFs in CRMs is often important for cooperative TF binding. By considering the order of a motif pair using the binomial distribution, with an FDR of 0.05, we found significant orders of 35693 motif pairs in 35182 motif modules (additional file 4). In these motif modules, the median of the percentage of CRMs with the defined order is 71.2%. The order of motif pairs in motif modules supports that these predicted motif modules may be biologically important and the order of TFBSs of different TFs may contribute to the efficiency of gene regulation. These defined orders between motifs will help future motif module identification. For instance, the motifs M00495 (BACH1) and M00926 (AP-1) have a preferred order, in which BACH1 binding sites are on the 5' of the AP-1 binding sites. For each of the 42 motif modules containing these two motifs, more than 94.6% of the CRMs have this order. The fact that this order is kept in such a high percentage of the CRMs of so many motif modules shows that most likely there is an order between the binding sites of the two TFs BACH1 and AP-1, although there is no literature support this order yet. Such a significant order found in this study may be applied to filter false positive predictions in future CRM predictions.

The distance between TFBSs within a CRM and the distance of the CRMs to the transcription start (stop) sites often affect the efficiency of gene regulation. We found that TFBSs of 9086692 motif pairs in 2857010 motif modules have specific preferred distance ranges. For instance, the TFBSs of the two motifs M00034 (P53) and M00469 (AP2alpha) prefer a distance between 6 bp and 55 bps in our predicted CRMs. It has been previously shown that AP2alpha physically interacts with P53 to regulate genes in cell growth and metastasis [45]. We also found that the CRMs of 1217409 motif modules have their distance ranges to the closest transcription start (stop) sites. For instance, the CRMs of the motif module "285236" mentioned in example 2 have a preferred distance range between 258 bp and 357 bp to the transcription start sites of their target genes, which further supports the function of this motif module. Furthermore, the CRMs of 210744, 1074481, 1085929 motif modules prefer to occur in the upstream, downstream and introns of their target genes, respectively.

The grammar defined by the order and the distance preference of the motif modules will help to discover new CRMs and filter false positives in future motif identification. Because of the predicted distance preference for a large number of motif modules and the limit of the additional files required by the journal, we can only show some results here. The detailed information can be found at http://www.cs.ucf.edu/~xiaoman/module1109.

## Discussion

We have developed a valuable method to predict motif modules and CRMs in the entire non-coding regions around human genes. In total, we have predicted 116226 CRMs and 3161839 motif modules, 90.8% of which are partially supported by various forms of functional evidence with an FDR of 0.05. Compared with 14 ChIP-seq datasets, on average, with an FDR of 0.01, 10.7% of the predicted TFBSs fell into the ChIP-seq peak regions and 69.6% of ChIP-seq peak regions containing multiple TFBSs overlap with our predicted CRMs. Comparing our predictions with the predicted CRMs in the previous study [24], our method shows at least comparable performance in identifying functional non-coding sequences.

Our method is based on local alignments and discontiguous sequence similarity. Unlike coding sequences, TFBSs in the non-coding sequences often cannot be aligned well with their corresponding TFBSs in other species. Trying to identify CRMs and motif modules from multiple alignments may miss many CRMs and motif modules. Moreover, differences among genome alignments generated by different methods affect the results [26]. We find that local alignments are better than multiple alignments for a detailed match of the TFBSs and for the identification of functional elements in orthologous sequences. Since often only the segments around TFBSs in a CRM are conserved, it is natural to consider discontiguous sequence similarity. To our knowledge, this is the first study that identifies CRMs in the entire human non-coding regions based on discontiguous sequence similarity.

A significant difference between our predicted CRMs and those found elsewhere is that our predicted CRMs share similar motif combinations with many other non-overlapping CRM regions. This recurrence of the same motif combination greatly decreases the chance that the motif instances of the motifs in these combinations co-occur in 1 kb long regions by chance. By comparing the predicted target genes with the gene sets in mSigDB, we indeed have shown that 90.8% of our motif modules are supported by at least one type of evidence.

In future studies, we will include more top 1 kb long regions such that the functional elements in the blocks cover a greater percentage of the human genome. Currently, although the total blocks cover about 5% of the human genome, the functional elements within these blocks cover a much smaller percentage of the genome. We will also incorporate other types of data besides sequence data to identify motif modules and CRMs. For instance, gene expression data and gene tissue specificity will definitely help to filter false positive predictions. Moreover, incorporating information from recent high throughput enhancer studies [44] should further improve the developed method. With these additions, we expect to identify more significant motif modules. Moreover, the sensitivity of all the predicted motif modules could be further improved.

## Conclusions

Our work provides a large-scale prediction of motif modules in mammals, and will facilitate the understanding of gene regulation in a systematic way.

## Methods

### Orthologous sequences, TRANSFAC motifs, and ChIP-seq data

We downloaded mammalian orthology information from the MGI database. We obtained 14961 orthologous gene groups containing one human gene and at least one rodent gene, composed of 14961, 13628, and 6991 genes from human, mouse, and rat, respectively. Based on the locations of genes in each species, we obtained the non-coding sequences for every gene. The non-coding sequence of a gene includes the upstream sequence from the closest codon of the 5' adjacent gene to the translational start site of the gene, the downstream sequence from the stop codon of the gene to the closest codon of the 3' adjacent gene, and the intron sequences within the gene. We downloaded all 522 vertebrate motifs from TRANSFAC 9.2. We downloaded all ChIP-seq data with restriction date until March 11, 2010 from http://hgdownload.cse.ucsc.edu/goldenPath/hg18/encodeDCC/wgEncodeYaleChIPseq/. We obtained 14 ChIP-seq datasets with narrow peak defined for the following 13 TFs: TCF7L2, c-Fos, c-Myc, STAT1, Max, SREBP2, E2F4, GATA-1, c-Jun, c-Myc, STAT2, NF-E2, and YY1. Details about the ChIP-seq data are provided in the additional file 1.

### Discontiguous sequence similarity and the identification of blocks

To define the discontiguous sequence similarity of two regions R and R', S(R, R'), we first applied a popular local alignment software package called CHAOS [46] for aligning R and R'. CHAOS generates local alignments by identifying similar k-mers in two sequences and then extending these k-mers [46]. We set the word length parameter to 6 bps and the degeneracy parameter to 0 when applying CHAOS. This is because the TFBSs are at least 6 bp long and we expect many functional 6-mers are exactly conserved in the non-coding sequences of orthologous genes between human and rodent. We considered the aligned pairs of segments output from CHAOS to be conserved segments. We defined S(R,R') as the sum of the CHAOS local alignment scores of all pairs of conserved segments within R and R'.

To identify the potential CRMs in the entire non-coding regions of the human genome, we implemented the following three-step procedure. First, we obtained conserved segments by applying CHAOS to the non-coding sequences of a human gene and one of its orthologs. Second, for every 1 kb long human region R, which starts from a conserved segment, we measured its conservation C(R). C(R) was calculated in the following way: Assume R is from the human gene H1. Assume H1 has mouse and rat orthologs M1 and R1. Assume R' and R'' are the 1 kb long regions in the non-coding sequences of M1 and R1, respectively, that have the highest discontiguous similarity scores with R among all 1 kb long regions in the corresponding species. Then C(R) = (S(R, R')+S(R,R''))/2. If there is no mouse or rat ortholog, then the conservation score is exactly equal to the similarity score of the existing orthologous pair (i.e. C(R) = S(R, R') or C(R) = S(R,R'')). Third, we identified the conservation score cutoff such that at most 5% of 1 kb long human regions have a conservation score larger than this cutoff. Finally, we obtained blocks by merging overlapping 1 kb long regions with conservation scores larger than this cutoff and removing the 3' sequences in these regions that do not contain any conserved segments.

### Identification of motif modules and their CRMs

To identify motif modules and CRMs, we first identified TFBS candidates. For each of the 522 vertebrate motifs, we defined a score cutoff such that the TFBSs of this motif occur in random sequences with a probability less than 0.0001. Random sequences were obtained by randomly picking 10000 non-coding genomic regions of 1 kb. With the cutoff of each motif, we scanned the conserved segments in the blocks and defined TFBS candidates as those segments with a score larger than the corresponding cutoff. Second, we identified frequent motif combinations from these TFBS candidates. Taking each block as a CRM candidate and the TFBS candidates in the block as a superset of the TFBSs of a motif module, we applied the FP-tree technique [30,31] to identify the motif module candidates. The FP-tree data structure and algorithm were originally developed as a database mining tool [31] to identify frequent combination of patterns in databases. Consider a database containing customer purchase information. With what frequency will a customer who buys item A also buys item B and C? The FP-tree data structures and algorithm can efficiently solve this type of problems. In our model, the 522 motifs correspond to items and blocks to customers. We used the FP-tree technique to output all frequent motif combinations with instances in at least 100 blocks. We chose 100 as the cutoff because this is the smallest cutoff our desktop computers can handle. Third, we justified the statistical significance of the frequent motif combinations and identified 3161839 motif modules. We used the Poisson clump heuristic to calculate the statistical significance of motif modules as in [32]. All frequent motif combinations with a Bonferroni corrected p-value less than 0.05 are output as motif modules. The blocks containing instances of these motif modules are output as CRMs. For a CRM, without specific descriptions, we defined the gene that is closest to this CRM as the target gene containing this CRM. That is, this gene is a target gene of the motif module.

### Overlap with functional evidence

We used the hypergeometric distribution to judge the significance of the overlap of target genes of a motif module with a pre-defined gene set. Consider a gene set that contains M genes and m out of these M genes are the target genes of the motif module under consideration. Assume this motif module contains n genes and there are N genes in the genome. Then the p-value is defined as $p(N,M,n,m)=1-\sum_{t=0}^{m-1}\frac{C_{M}^{t}C_{N-M}^{n-t}}{C_{N}^{M}}$. With these p-values, we used the p.adjust function in the R package to output significant motif modules with an FDR of 0.01 and 0.05, respectively.

### Order and distance preference of motif modules

For every motif pair in each motif module, we determined the significance of the order preference in the following way: Assume we were considering a motif pair (A, B) in a motif module with n target genes. Let *m *be the number of times the TFBSs of A were at the 5' of the TFBSs of B. Then we assigned an order significance p-value as $p(n,m)=1-\sum_{t=0}^{m-1}\frac{C_{n}^{i}}{2^{n}}$ if m>n/2 or $p(n,m)=1-\sum_{t=m+1}^{n}\frac{C_{n}^{i}}{2^{n}}$ if *m*< = *n/2*, where $C_{n}^{i}$ is the combinatorial number of *n *choose *i*. We then output all motif modules with q-value less than 0.01 as motif pairs with significant orders.

For every motif pair in each motif module, we determined the distance preference with a Poisson distribution. We first calculated the distances of TFBS of two motifs in every CRM. We then divided these distances into bins, with every 50 bp as a bin. Then we calculated the rate parameter of the Poisson distribution as the average counts of the CRMs within each bin. Finally, we calculated the p-value of distance preference based on the Poisson distribution with the estimated rate parameter. We did similar analyses to determine the distance preference of CRMs of a motif module relative to the transcription start (stop) sites. For strand preference of motif pairs within a motif module, we performed analysis similar to the analysis of the order preference. Based on these p-values, we output motif pairs with a Bonferroni corrected p-value less than 0.01 and 0.05 as significant ones.

## Abbreviations

CE: composite elements; CRM: cis-regulatory module; FDR: false discovery rate; FP-tree: frequent pattern tree; MGI: mouse genome informatics; TF: transcription factor; TFBS: transcription factor binding site.

## Authors' contributions

HH and XL designed the research. XC, LH, NS and MD collected the data and analyzed the data. XC, LH, NS, HH, MD and XL wrote the manuscript. All authors read and approved the final manuscript.

## Supplementary Material

Additional file 1 **The readme file of the ChIP-seq data analysis**. This file describes the ChIP-seq data used and the analysis of ChIP-seq data performed. Click here for file

Additional file 2 **Comparison of ChIP-seq data with predicted TFBSs of individual motifs**. This file describes the results of the comparisons of the ChIP-seq data with the predicted TFBSs for individual motifs. Click here for file

Additional file 3 **Comparison of the predicted motif pairs with pairs of ChIP-seq experiments**. This file describes the results of the comparison of the predicted motif pairs with pairs of ChIP-seq experiments. Click here for file

Additional file 4 **Motif order preference**. This file describes the order preference among motifs in the predicted motif modules. Click here for file
