# Supplementary material for: Systematic identification of conserved motif modules in the human genome
Source: BMC Genomics. 2010 Oct 14;11:567. doi: 10.1186/1471-2164-11-567 (PMC3091716; doi:10.1186/1471-2164-11-567)
Supplement: Additional file 1 — The readme file of the ChIP-seq data analysis. This file describes the ChIP-seq data used and the analysis of ChIP-seq data performed. [file 1471-2164-11-567-S1.DOC]

**ChIP-seq data**

We downloaded all ChIP-seq data with defined narrow peaks and with restriction date until March 11, 2010 from:

<http://hgdownload.cse.ucsc.edu/goldenPath/hg18/encodeDCC/wgEncodeYaleChIPseq/>.

For each ChIP-seq dataset, we used the cutoff FDR<0.01 (q>2) to extract peaks from the narrow-peak data file. Meanwhile, we also overlapped these peaks with the human non-coding regions we used to predict motif modules. The peaks that do not have any overlap with our regions were eliminated. The number of the remaining peaks is described in the column D of the additional file 2. The 14 ChIP-seq datasets are described in the column B of the additional file 2.

These ChIP-seq data correspond to 30 motifs. The 30 motifs are in the following: M00037, M00059,M00069,M00118,M00123,M00127,M00128,M00172,M00203,M00223,M00322, M00346,M00492,M00496,M00517,M00615,M00671,M00776,M00777,M00789,M0079, M00799, M00803, M00919, M00920, M00924,M00926,M00983,M01034, M01035.

**The threshold to define the binding sites of a motif**

For every motif, we collected its predicted binding sites from all significant motif modules. Then we got the sequence of these binding sites. We defined the score of a binding site as:


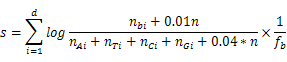


where d is the length of this motif, $n_{bi}$ is the count of nucleotide b at position i in this binding site, $f_{b}$ is the background distribution of the nucleotide b, and
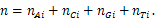
Here $f_{A}$=$f_{T}$= 0.2960936, $f_{C}$=$f_{G}$= 0.2039064, which is the nucleotide frequency in human non-coding sequences. Some of the predicted binding sites are within ChIP-seq peaks. For every motif, the minimal score of the predicted binding sites within some ChIP-seq peaks was defined as the threshold (namely T) of this motif. If a segment has a score larger than this threshold, this segment will be called as a putative binding site of this motif under consideration.

**Compare predicted binding sites of individual motifs with individual ChIP-seq experiments**

For every motif, we extracted the sequences of all peaks, and calculated the score of every segment and its reverse complement segment in these sequences similarly as what we did for the predicted binding sites. The number of peaks that have at least one binding site with the score larger than T was defined as K. These binding sites were thought as true binding sites of this motif, since they had a high score and they show binding activities in the ChIP-seq experiment. For every motif, we also define the number of predicted binding sites as L; the number of overlapped binding sites with ChIP-seq peaks as N; and the number of ChIP-seq peaks as M.

From the above description, the number of real binding sites is K, and the number of predicted binding sites is L. The number of overlapped binding sites N is the number of true predictions. So precision=N/L, recall= N/K.

There are 12 columns in the additional file 2 for the comparison of binding sites of individual motifs with ChIP-seq experiments.

A: motif name

B: the name of the original ChIP-seq data

C: the corresponding transcription factor

D: the number of peaks within the human regions we used.

E: the number of overlapped binding sites of the motif (N)

F: the number of predicted binding sites of the motif (L)

G: the threshold (the minimal score of all binding sites of the motif) (T)

H: the number of real binding site (=the number of peaks with binding site scores larger than the threshold)(K)

I: precision=N/L

J: recall=N/K

K: precision/recall

**Compare CRMs of motif pairs with a pair of ChIP-seq experiments**

There are 32 predicted motif pairs in our predictions that are composed of the 30 motifs mentioned above. Among them, 8 motif pairs correspond to the same transcription factors. The remaining 24 motif pairs are composed of motifs from different transcription factors. We thus compared the predicted CRMs of these 24 motif pairs with the corresponding pairs of ChIP-seq data.

We have defined real binding sites above (column H of the additional file 2). For a motif pair, we define the real CRMs as the overlapped peaks for the two transcription factors. That is, two peaks defined in column H of the additional file 2 for the two transcription factors must overlap in order to be claimed as a real CRM. Assume the number of the real CRMs is m. We compared these real CRMs with our predicted CRMs for the same motif pairs, and defined the overlapped CRMs as the true predictions. Assume the number of overlapped CRMs is n. Then n/m defines the recall of the method at the motif pair level. The result is showed in the additional file 3.

There are 16 columns in the additional file 3. The first 6 columns are the motif, the corresponding factor, the corresponding ChIP-seq data, the number of predicted binding sites, the number of peaks in the ChIP-seq data, and the number of overlapped binding sites, for the first motif in the motif pair. The next 6 columns are for the second motif in the motif pair, in the same format as the first 6 columns. The 13-th column is the number of predicted CRMs containing the motif pairs, the 14-the column is the number of "true CRMs"(m), the 15-the column is the number of true CRMs overlapping with the predicted CRMs (n), and the last column is n/m.
